# Supplementary material for: Content-rich biological network constructed by mining PubMed abstracts
Source: BMC Bioinformatics. 2004 Oct 8;5:147. doi: 10.1186/1471-2105-5-147 (PMC528731; doi:10.1186/1471-2105-5-147)
Supplement: Additional File 2 — The original results of the above study (non-essential files are deleted to keep the file size under the limit set by BMC bioinformatics). [file 1471-2105-5-147-S2.bz2 › chilibotAdditionalFile2/dip40/559ID10395669E10919/html/IFNAR2_IFNB1.html]

 


 **IFNAR2** and **IFNB1** 
  
Found 32 abstracts in PubMed, retrieved 32.  
 

 What does Google say? 
 PDF only 
| .edu only 

---

**Interactive relationship** (e.g. stimulation, inhibition, etc)

**Neutral relationship**- The results suggest that the binding energy from interaction of IFN beta  [ **IFNB1** ]  with  **ifnar2**  serves mainly to stabilize the bound IFN receptor complex.  Ref: 10704203 Biochemistry, 2000
- Formation of a uniquely stable type I interferon receptor  [ **IFNAR2** ]  complex by interferon beta  [ **IFNB1** ]  is dependent upon particular interactions between interferon beta  [ **IFNB1** ]  and its receptor and independent of tyrosine phosphorylation.  Ref: 10049744 Biochem Biophys Res Commun, 1999
- Both IFNAR1 and  **IFNAR2** .2 undergo tyrosine phosphorylation upon induction by either IFN alpha or IFN beta  [ **IFNB1** ] .  Ref: 8969169 J Biol Chem, 1996
- In contrast, IFN alpha and IFN beta  [ **IFNB1** ]  are ligands for the anti inflammatory type I interferon receptor  [ **IFNAR2** ]  and are beneficial in some but not all patients with MS.  Ref: 10204571 J Leukoc Biol, 1999
- The Type I interferon receptor  [ **IFNAR2** ]  IFN alpha R interacts with all IFN alpha s, IFN beta  [ **IFNB1** ]  and IFN omega, and seems to be a multisubunit receptor.  Ref: 8070579 FEBS Lett, 1994
- Interferon beta  [ **IFNB1** ]  selectively induces the interaction of the alpha and betaL subunits of the type I interferon receptor  [ **IFNAR2** ] .  Ref: 8798579 J Biol Chem, 1996
- The ability to produce the IFN beta  [ **IFNB1** ]  1a  **IFNAR2**  and IFN beta  [ **IFNB1** ]  1a IFNAR1  **IFNAR2**  complexes make them attractive candidates for X ray crystallography studies aimed at determining the molecular interactions between IFN beta  [ **IFNB1** ]  1a and its receptor.  Ref: 10493588 Protein Sci, 1999
- A mutant clone, TMK, fully resistant to alpha interferon with good sensitivity to beta interferon  [ **IFNB1** ] , was characterized in detail and compared with U5A cells complemented with wild type  **ifnar2**  and also with Tyk2 deficient 11.1 cells, which exhibit a similar alpha unresponsive phenotype with a partial beta interferon  [ **IFNB1** ]  response.  Ref: 9737924 J Mol Biol, 1998
- The human type I interferons, including at least 12 IFN alphas, IFN beta  [ **IFNB1** ]  and IFN omega, bind to a receptor IFNAR composed of at least two transmembrane subunits, IFNAR 1 and IFNAR 2  [ **IFNAR2** ] .  Ref: 9119063 FEBS Lett, 1997
- The human type I interferons, IFN alpha, IFN beta  [ **IFNB1** ] , and IFN omega, induce somewhat different cellular effects but act through a common receptor complex, IFNAR, composed of subunits IFNAR 1 and IFNAR 2  [ **IFNAR2** ] .  Ref: 11278538 J Biol Chem, 2001
- cDNAs encoding the Type I interferon receptor  [ **IFNAR2** ]  subunit were amplified by polymerase chain reaction, using as template RNA isolated from three melanoma cell lines displaying greater than 100 fold range in their sensitivity to the antiproliferative effects of IFN beta  [ **IFNB1** ] .  Ref: 7951047 Biochem Mol Biol Int, 1994
- However, two mAb that bound to sites remote from the IFNAR 2  [ **IFNAR2** ]  binding site on IFN beta  [ **IFNB1** ]  also inhibited IFN beta  [ **IFNB1** ]  IFNAR 2  [ **IFNAR2** ]  complex formation and demonstrated potent neutralizing activity.  Ref: 11747625 J Interferon Cytokine Res, 2001
- Immunoprecipitation experiments demonstrated that  **IFNAR2** .2 is present in Daudi cells as a cell surface protein of approximately 90 100 kDa, which is tyrosine phosphorylated and associated with IFNAR1, upon stimulation of cells with IFN beta  [ **IFNB1** ] .  Ref: 8969169 J Biol Chem, 1996

**Non-interactive relationship** (e.g. studied together, co-existance, homology, etc.)

- Differential tyrosine phosphorylation of the IFNAR chain of the type I interferon receptor  [ **IFNAR2** ]  and of an associated surface protein in response to IFN alpha and IFN beta  [ **IFNB1** ] .  Ref: 7813427 EMBO J, 1994
- Two of these inhibited IFN beta  [ **IFNB1** ]  IFNAR 2  [ **IFNAR2** ]  complex formation, suggesting that steric hindrance of receptor binding constitutes their mechanism of neutralization.  Ref: 11747625 J Interferon Cytokine Res, 2001
- However, a stable binary complex formed between IFN beta  [ **IFNB1** ]  1a and  **IFNAR2** .  Ref: 10493588 Protein Sci, 1999
- Gel filtration of a mixture of IFNAR1 and the IFN beta  [ **IFNB1** ]  1a  **IFNAR2**  complex indicated that the three proteins formed a stable ternary complex.  Ref: 10493588 Protein Sci, 1999
- The extracellular portions of the chains that comprise the human type I interferon receptor  [ **IFNAR2** ] , IFNAR1 and  **IFNAR2** , have been expressed and purified as recombinant soluble His tagged proteins, and their interactions with each other and with human interferon beta  [ **IFNB1** ]  1a IFN beta  [ **IFNB1** ]  1a were studied by gel filtration and by cross linking.  Ref: 10493588 Protein Sci, 1999
- iii beta interferon  [ **IFNB1** ]  with  **ifnar2**  alone.  Ref: 9737924 J Mol Biol, 1998
- For example, IFN beta  [ **IFNB1** ] , but not IFN alpha, induces the association of tyrosine phosphorylated receptor components ifnar1 and  **ifnar2** , and has activity in cells lacking the IFN receptor associated, Janus kinase tyk2.  Ref: 9525899 J Biol Chem, 1998
- Therefore, it is unclear as to why  **IFNAR2** .2 is not detectable in IFNAR1 immunoprecipitates in IFN beta  [ **IFNB1** ]  treated cells.  Ref: 8969169 J Biol Chem, 1996
- We used specific antibodies recognizing the receptor 1 IFNAR1 and the recently cloned receptor 2.2  **IFNAR2** .2 chains of the human type I interferon receptor  [ **IFNAR2** ]  complex to demonstrate that the interferon beta  [ **IFNB1** ]  IFN beta  [ **IFNB1** ]  specific receptor associated phosphoprotein is  **IFNAR2** .2 and not an unknown or additional receptor component.  Ref: 8969169 J Biol Chem, 1996
- A panel of monoclonal antibodies mAb derived against human interferon alpha beta receptor 2  [ **IFNAR2** ]  IFNAR 2  [ **IFNAR2** ]  was evaluated for their ability to antagonize the biologic effects of type 1 interferons IFN alpha1, IFN alpha2a, and IFN beta  [ **IFNB1** ] .  Ref: 11096454 J Interferon Cytokine Res, 2000
- These data suggest that, although IFN alpha and IFN beta  [ **IFNB1** ]  may utilize similar receptor chains, they interact with IFNAR1 and  **IFNAR2** .2 in different ways.  Ref: 8969169 J Biol Chem, 1996
- Analysis of ternary complex formation using various molar excesses of IFNAR1 and the IFN beta  [ **IFNB1** ]  1a  **IFNAR2**  complex indicated that the ternary complex had a 1 1 1 stoichiometry, and reducing SDS PAGE of the ternary complex treated with DSG indicated that the major cross linked species had an apparent Mr consistent with the sum of its three individual components.  Ref: 10493588 Protein Sci, 1999
- Abolition of binding to  **ifnar2**  Fc for mutants A2, AB1, AB2, and E established that the  **ifnar2**  binding site on IFN beta  [ **IFNB1** ]  comprises parts of the A helix, the AB loop, and the E helix.  Ref: 10704203 Biochemistry, 2000
- Analysis of binary complex formation using various molar excesses of IFN beta  [ **IFNB1** ]  1a and  **IFNAR2**  indicated that the complex had a 1 1 stoichiometry, and reducing SDS PAGE of the binary complex treated with the cross linking reagent dissucinimidyl glutarate DSG indicated that the major cross linked species had an apparent Mr consistent with the sum of its two individual components.  Ref: 10493588 Protein Sci, 1999
- We conclude that the ternary complex forms by the sequential association of IFN beta  [ **IFNB1** ]  1a with  **IFNAR2** , followed by the association of IFNAR1 with the preformed binary complex.  Ref: 10493588 Protein Sci, 1999
- By gel filtration, no stable binary complexes between IFN beta  [ **IFNB1** ]  1a and IFNAR1, or between IFNAR1 and  **IFNAR2**  were detected.  Ref: 10493588 Protein Sci, 1999
- Using anti receptor antibodies and mutant forms of beta interferon  [ **IFNB1** ] , three distinct modes of ligand interaction could be discerned i alpha interferon with ifnar1 and  **ifnar2** .  Ref: 9737924 J Mol Biol, 1998
- ii beta interferon  [ **IFNB1** ]  with ifnar1 and  **ifnar2** .  Ref: 9737924 J Mol Biol, 1998
- 253J B V IFN R  [ **IFNAR2** ]  cells resistant to antiproliferative effects of IFN alpha or IFN beta  [ **IFNB1** ]  were implanted into the bladder wall of nude mice.  Ref: 10537335 Clin Cancer Res, 1999
